# Research Progress on Biological Accumulation, Detection and Inactivation Technologies of Norovirus in Oysters

**DOI:** 10.3390/foods12213891

**Published:** 2023-10-24

**Authors:** Yiqiang Sun, Meina Liang, Feng Zhao, Laijin Su

**Affiliations:** 1 College of Life and Environmental Science, Wenzhou University, Wenzhou 325035, China; sunyq@stu.wzu.edu.cn (Y.S.); lmn19818149332@163.com (M.L.); 2 Zhejiang Provincial Key Laboratory for Water Environment and Marine Biological Resources Protection, Wenzhou University, Wenzhou 325035, China; 3 College of Biology and Food Engineering, Chongqing Three Gorges University, Chongqing 404100, China; zhaof_cn@163.com

**Keywords:** norovirus, bioaccumulation, oysters, detection methods, inactivation methods

## Abstract

Noroviruses (NoVs) are major foodborne pathogens that cause acute gastroenteritis. Oysters are significant carriers of this pathogen, and disease transmission from the consumption of NoVs-infected oysters occurs worldwide. The review discusses the mechanism of NoVs bioaccumulation in oysters, particularly the binding of histo-blood group antigen-like (HBGA-like) molecules to NoVs in oysters. The review explores the factors that influence NoVs bioaccumulation in oysters, including temperature, precipitation and water contamination. The review also discusses the detection methods of NoVs in live oysters and analyzes the inactivation effects of high hydrostatic pressure, irradiation treatment and plasma treatment on NoVs. These non-thermal processing treatments can remove NoVs efficiently while retaining the original flavor of oysters. However, further research is needed to reduce the cost of these technologies to achieve large-scale commercial applications. The review aims to provide novel insights to reduce the bioaccumulation of NoVs in oysters and serve as a reference for the development of new, rapid and effective methods for detecting and inactivating NoVs in live oysters.

## 1. Introduction

Noroviruses (NoVs) belong to the Caliciviridae family and are envelope-free positive-sense single-stranded RNA viruses [1]. NoVs contain three open reading frames (ORFs): ORF1, which encodes the primary nonstructural protein of the virus; ORF2, which encodes the primary structural protein of the virus (capsid protein, VP1); and ORF3, which encodes the secondary structural protein (VP2) [2]. Based on the complete VP1 gene, NoVs can be divided into five genomes (GI–GV) [3]. Chhabra et al. further expanded the number of NoV genomes to 10 (GI–GX) based on the diversity of VP1 amino acid sequences [4]. NoVs are the most common pathogenic cause of global outbreaks of acute gastroenteritis, with common symptoms, such as vomiting, diarrhea and stomach cramps [5,6]. NoVs are mainly transmitted via the fecal–oral route and have a high incidence in autumn and winter [7,8]. Human NoVs (HuNoVs) include GI, GII and GIV, with GII being the most common [9]. NoVs cause acute gastroenteritis outbreaks as they are usually associated with foodborne and waterborne transmission and have high environmental resistance and pathogenicity (only 18 virus particles are required to cause the disease) due to the lack of vaccines and specific drugs against them [10].

In recent years, cases of foodborne disease caused by NoVs have been reported in many countries, including the United States, Japan and the United Kingdom, mostly due to consumption of raw or inadequately cooked NoV-contaminated shellfish [11,12,13]. Japan experienced a large outbreak of NoVs in 2006 [14]. After several investigations, it was concluded that this outbreak was caused by the consumption of NoV-contaminated shellfish. Thebault et al. modeled and analyzed NoV transmission events in France and found that they were highly associated with oyster consumption [15]. Oysters are typically filter feeders, and plankton, microalgae and viruses in seawater enter their filter-feeding system during feeding. NoVs often bioaccumulate in oysters through filter feeding, and this accumulation can increase the concentration of NoVs in oysters by tens to thousands of times that in the environment [16,17]. However, the NoVs that accumulate in oysters are often difficult to excrete during metabolic processes or purification [18].

Histo-blood group antigens (HBGAs) are the target receptors for NoVs. A complex carbohydrate present on the virus surface interacts with the fucose region of HBGAs [5]. Studies have confirmed the presence of multiple HBGA-like molecules in oyster intestinal tissues, to which various NoVs can specifically bind and bioaccumulate [19]. Further studies have confirmed that the seasonally dependent expression levels of NoV ligands in oysters were consistent with the bioaccumulation efficiency of NoVs in oysters [20,21], which may be one of the reasons for the high prevalence of NoV infections in autumn and winter. Lowther et al. investigated NoVs contamination in two oyster farming areas in the UK for 31 consecutive months and found that the detection rate of NoVs was approximately 17 times higher from November to March each year than that in other months [22]. Some researchers have suggested that this might be due to the fact that oysters secrete certain substances that favor the bioaccumulation of NoVs under low-temperature conditions [23].

Understanding the mechanisms of virus bioaccumulation in food matrices and developing more advanced assays to detect virus concentrations in matrices can provide prerequisites for subsequent efficient virus inactivation, thereby reducing the risk of viral transmission and maintaining food safety. Therefore, the review will discuss the mechanism of bioaccumulation of NoVs in oysters and the factors influencing accumulation and summarize the advantages and disadvantages of the main NoVs detection methods available today, while exploring the potential of CRISPR/Cas technology as the next generation of mainstream virus detection tool. In addition, the review also discusses the effects of non-thermal processing (NTP) treatments on food matrices and virus inactivation, focusing on high hydrostatic pressure (HHP) treatments that rely on the application of pressure to inactivate viruses.

## 2. NoVs Contamination in Oysters

**Figure 1:**
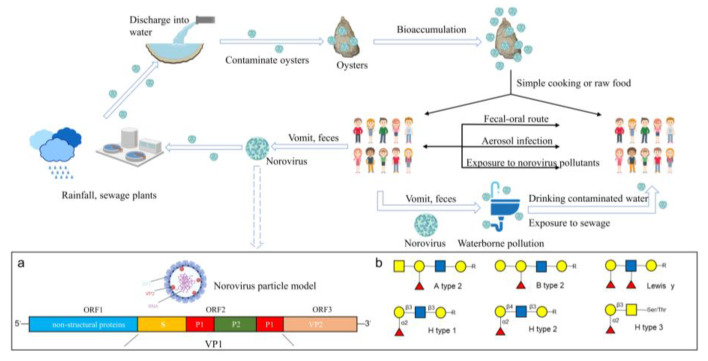
Schematic diagram of NoVs propagation. After humans are infected with NoVs, they are carried in feces and vomit, which are discharged via precipitation and sewage treatment plants into the waters where oysters thrive. NoVs enter oysters and bioaccumulate, and the risk of transmitting NoVs to humans after simple processing or direct raw food consumption remains. In addition, NoVs can also contaminate drinking water sources and form a human-to-human transmission. (**a**) NoV particle model and genome structure of NoV. (**b**) Structural diagram of HBGAs. Yellow circle: galactose (Gal); red triangle: fucose (Fuc); blue square: N-acetylglucosamine (GlcNAc); yellow square: glucose (Glc).

Oysters are globally important marine food resources and a major vector for foodborne viruses [24]. NoVs have been detected in bivalve shellfish, particularly oysters, in numerous countries and regions (Table 1). The concentrations of NoVs detected in shellfish correlate with the number of local NoV outbreaks [25]. For example, in eight NoV outbreak investigations in the United States from 2009 to 2014, NoVs were detected in all bivalve shellfish samples from outbreak areas and showed 100% homology with clinical strains [26]. Humans who consume oysters contaminated with NoVs are prone to vomiting, diarrhea and other symptoms [10]. Feces, vomit and untreated sewage containing large amounts of NoVs may contaminate drinking water sources or be discharged into seawater through stormwater and sewage treatment plants, increasing the concentration of NoVs in seawater and recontaminating oysters, creating a cycle of NoVs in humans, the aquatic environment and oysters. In addition, through the fecal–oral route, aerosol infection and exposure to pollutants, NoVs can also form human-to-human transmission (Figure 1).

## 3. Bioaccumulation Mechanism of NoVs in Oysters

### 3.1. Oyster Feeding Mechanism

Oysters are typical filter-feeding bivalves, which draw seawater into their gills during feeding. During feeding, a large number of suspended particles are transported together into the digestive tract, with some of the particles being excreted and others being absorbed by phagocytic blood cells. NoVs usually exist in the water column as adsorbed particles and are absorbed along with other particles by blood cells [36]. Blood cells contain acidic vesicles, and whether viruses can survive in blood cells depends on their stability in acidic conditions [37]. NoVs are highly resistant to acidic environments and can therefore remain in blood cells for a long time and are subsequently transported to the peri-intestinal tissues of oysters [38]. NoVs then bind to HBGA-like molecules in the gastrointestinal tract epithelium, leading to their bioaccumulation [39].

Although all bivalves can be contaminated by NoVs during filter feeding, oysters play a more important role in the transmission of NoVs than other bivalves, possibly due to the mode of consumption (raw or lightly cooked), the proximity of their habitat to sewage outfalls, or the ability of NoVs to specifically bind to HBGA-like molecules, which are difficult to remove from oysters [40,41,42].

### 3.2. Bioaccumulation and Persistence of NoVs in Oysters

The bioaccumulation of NoVs in oysters is correlated with their genotypes. A study of NoV contamination in an oyster farming area in France reported that the percentage of NoV GI- and GII-positive samples was 59% and 70%, respectively, in 17 samples collected during the first week, and decreased to 41% and 17%, respectively, in 17 samples collected after 3 weeks [43]. The decrease in GI-positive samples was significantly smaller than that in GII-positive samples, which indicated that GI had a stronger persistence in oysters than GII, probably because the binding strength to HBGA-like molecules in vivo is higher for GI than that for GII. Another study based on RT-qPCR determined the binding of NoVs to oyster tissue by microwell plates coated with oyster gut homogenates, and the results showed that the highest binding rate was found with NoV GI.3 and oysters (97.3%), whereas 35.1% of NoV GII.4 bound to oysters [16]. Therefore, GI was detected frequently in NoV outbreaks triggered by oysters. Molecular dynamics membrane simulation data showed that the high binding strength of NoVs to HBGA-like molecules was due to fully exposed (1,2)-linked α-L-Fucp and (1,4)-linked α-L-Fucp residues, and weaker binding strengths or an inability to bind was due to the very limited accessibility of (1,3)-linked α-L-Fucp residues when glycolipids were embedded in phospholipid membranes [44]. Within the same genome, different genotypes of NoVs exhibit different degrees of bioaccumulation efficiency in oysters. For example, NoV GII.4 and GII.3 have similar binding patterns to digestive tissues, gills and mantle, but the bioaccumulation efficiency varies greatly. GII.3 was effectively bioaccumulated by oysters. In contrast, the bioaccumulation level of GII.4 was very poor, and the concentration rate in oysters was less than 0.01% [21].

Several studies investigating the presence of NoV-specific ligands in oysters have found that oysters can selectively bioaccumulate NoVs via the specific binding of HBGA-like molecules to NoVs in vivo. For example, NoV GI.1 accumulates in the midgut and digestive diverticulum of oysters, but not in other tissues [45]. GII.4 accumulates in multiple tissues, including the midgut, digestive diverticula, gills and mantle [40,46]. Type A HBGA-like molecules are prevalent in the digestive tissue, gills and mantle of oysters, whereas type H1 and Lewis b HBGA-like molecules are mainly expressed in digestive tissues [19,47]. Using validation tests with monoclonal antibodies and particles of NoVs with a mutant capsid, Le Guyader et al. confirmed that NoV GI.1 bound to oyster tissues via type A HBGA-like molecules, similar to the binding of NoVs to HBGAs in human epithelial cells [45]. The binding of NoV GII to oyster digestive tissues also occurs via type A HBGA-like molecules, whilst binding to the gills and coat membranes occurs via sialic acid residues [20,21]. To assess the effect of ligands on the bioaccumulation of NoVs in oysters, Maalouf et al. selected three strains of NoVs (GI.1, GII.3 and GII.4) and compared their bioaccumulation efficiencies, tissue distributions and seasonal influences [21]. The findings showed that GI.1 bound to digestive tissues via type A HBGA-like molecules but hardly bound to a few other tissues and the concentration was negligible. For example, the concentration of GI.1 in the gill and coat membrane after 24 h was 1000-fold different from that in digestive tissues. The bioaccumulation efficiency of GI.1 in the digestive tissues of oysters is consistent with the seasonally dependent expression levels of the ligand [20]. In specific months, the level of GI.1 in oysters is more active, and bioaccumulation efficiency also significantly increases. In contrast, the bioaccumulation efficiency of GII.4 in oysters is low regardless of the month and shows a different tissue distribution. After the GII.4 strain enters the oyster, it preferentially accumulates in the digestive tissues within 24 h. However, most of the virus is then transferred to the gills and mantle by binding to sialic acid residues, which may prevent subsequent viral particles from entering the oyster’s digestive tissues [21]. The concentration efficiency of NoV GI in oysters is superior to that of GII. To bioaccumulate 1 viral RNA copy/g oyster tissue, the concentration of NoV GI in water needs to be 30 viral RNA copies/L water, whereas it needs to be 1200 viral RNA copies/L water for GII [48]. The result provided further evidence for NoV GI to be a dominant strain in oysters.

### 3.3. Inhibition of NoVs Binding to HBGA-Like Molecules

Inhibiting the interactions between NoVs and HBGA-like molecules in oysters is a feasible method for preventing novel transmission [49]. Some bacteria exhibit strong anti-NoVs activity and can be used to reduce NoVs infection in oysters [50,51]. According to Gorji et al., *Bifidobacterium bifidum* JCM 1254 secreted a rockulosidase that could hydrolyze certain HBGAs bound to NoVs in the digestive tissue of oysters [52]. Therefore, it was proposed that the discovery or artificial modification of specific active rockulosidase-secreting strains and their application would have great potential for reducing the risk of NoVs outbreaks. Li et al. screened one of the 122 Lactobacillus isolates for the most potent murine NoV (MNV) antagonist, *Limosilactobacillus fermentum* PV22, which was observed to reduce viral titers by 2.23 ± 0.38 (logarithmic) in 5 min [51]. Genome mining revealed that the antiviral activity of this strain was due to the synthesis of γ-aminobutyric acid. The results of this study suggest that the use of bacteria with anti-NoVs activity could be an effective method for preventing NoVs outbreaks.

## 4. Potential Factors Affecting the Bioaccumulation of NoVs in Oysters

Several environmental factors, including but not limited to temperature and precipitation, have been found to be associated with the bioaccumulation of NoVs in oysters [53]. Water pollution in oyster farming areas also contributes to this bioaccumulation [54].

### 4.1. Temperature and Precipitation

NoVs outbreaks have obvious seasonal variations, and NoVs contamination levels are usually significantly higher in autumn and winter than those in spring and summer, probably due to the strong viability of NoVs at low temperatures, while the intensity of ultraviolet radiation in autumn and winter is weak [55]. In bivalves, the elimination of accumulated NoVs is often difficult to achieve when the ambient temperature is low, thus enhancing their bioaccumulation. According to Seo et al., the detection rates of NoV GII in oysters in Korea were 25.0% and 13.6% in winter and summer, respectively [32]. Data from Rince et al. showed that the detection rates of NoVs in French shellfish were 64.2% and 15.4% in winter and spring, respectively, whereas NoVs were not detected in summer when the temperature was the highest [56]. To compare the persistence of NoVs in oysters at different temperatures, Choi et al. placed oysters in seawater at 7 °C, 15 °C and 25 °C [57]. NoVs could be detected in oysters kept at 25 °C for 4 weeks, whereas they were detected in oysters kept at 7 °C and 15 °C for 6 weeks. This result confirmed that NoVs can survive in oysters for a longer duration at lower temperatures. However, the NoVs detection rate was not higher in the low-temperature environments than in the high-temperature ones. Polo et al. examined NoVs in bivalve shellfish samples collected from Galicia, Spain, and found that NoV GI and GII were detected at rates of 44.4% and 33.3%, respectively, in the warm season, but only at 20.7% and 18.4%, respectively, in the cold season [58]. The shellfish collection site used in this study has special oceanographic conditions that generate upwelling during spring and summer. Upwelling increases ocean productivity and facilitates plankton growth and reproduction. This plankton can maintain the water temperature within a stable range, whereas NoVs in seawater bind to plankton particles via electrostatic interactions and enter the digestive system of oysters during the oyster feeding process, thereby increasing the efficiency of NoVs bioaccumulation in oysters [59,60]. Precipitation is also an important factor affecting NoVs bioaccumulation. Investigations have shown that when rainfall exceeds 140 mm, oysters within 2–8 km of the outfall may test positive for NoVs. Precipitation exceeding the maximum treatment capacity of urban wastewater treatment systems can cause a rapid decrease in wastewater treatment efficiency and can even lead to the discharge of wastewater into water bodies before any treatment [61,62]. Therefore, excreta containing NoVs can easily enter the waters where oysters grow, resulting in NoVs accumulation.

### 4.2. Water Pollution

Oyster-growing waters become contaminated with NoVs in two ways: sewage discharge and vessel discharge. Sewage discharge, including that from sewage treatment plants, septic tanks, and their associated overflows, carries NoVs into the waters where oysters are located [63,64], increasing the risk of oysters becoming infected with them. Some wastewater treatment processes can reduce the level of NoVs in the effluent; however, due to the high resistance of NoVs to the external environment, some remain in the effluent even after the treatment [65]. For example, after treatment with the common activated sludge method, NoV GI levels in wastewater were reduced by 3.1 log_10_ and GII by 2.3 log_10_ [66]. Ibrahim et al. treated Tunisian municipal domestic wastewater with the activated sludge method and UV-C_254_ and then examined NoV GI and GII concentrations; the results showed that the removal effect of UV-C_254_ on NoV GI and GII was better than that of the activated sludge method [67]. Prado et al. used three wastewater treatment methods, namely activated sludge, membrane bioreactor/reverse osmosis and sand-anthracite filtration, to remove NoVs from wastewater [68]. The results showed that, of these three methods, membrane bioreactor/reverse osmosis was the most effective, with no NoVs detected in the resulting reuse water, followed by the sand-anthracite filter with removal efficiencies of 1.1–1.6 log_10_ (GI) and 0.7–1.6 log_10_ (GII); activated sludge had the worst removal efficiency, ranging from 0.3 to 0.8 log_10_ for GI and 0.4 to 1.4 log_10_ for GII. Therefore, membrane bioreactor/reverse osmosis is highly suitable for reclaimed water production and can effectively reduce pathogens in water. In waters located far from sewage outfalls, oysters may get contaminated with NoVs from vessel discharge. It is estimated that the concentration of NoVs discharged by large cruise ships during their voyages can reach 10^4^ viruses/L, and this NoV-containing discharge is released directly into seawater, expanding the population of oysters infected with NoVs [54]. Preventing oysters from coming into contact with contaminated seawater is a critical step in preventing them from triggering NoVs outbreaks. Prohibiting oyster fishing near sewage outfalls is the most direct approach. However, if the effluent quality continues to decrease, it will cause problems such as the increasing scope of the no-take zone and damage to the interests of fishermen; therefore, it is necessary to reduce the pollution from the source of effluent discharge. The development of relevant discharge standards and improvement of sewage treatment processes and other environmental factors can help develop effective programs to reduce the risk of oysters being infected with NoVs.

## 5. Detection Methods

The reverse transcription quantitative PCR(RT-qPCR) technique is a widely used method for detecting NoVs in bivalves and is considered the gold standard [69,70]. It uses a fluorescent-labeled probe to confirm the presence of a specific target and has the advantages of speed, sensitivity, and specificity in the detection of NoVs [71]. However, RT-qPCR requires expensive laboratory instruments and professional operators and is therefore not suitable for small clinics or community health settings. Another important limitation of RT-qPCR is that it cannot discriminate between infectious and non-infectious NoVs [72], which may lead to incorrect estimation of infectious NoVs. Pretreatments, such as enzymatic RNase and photo-activatable dyes (PMA, PMAxx), performed prior to molecular detection can reduce the effect of non-infectious viral RNA on detection results to some extent [73]. Pretreatment with RNase is effective in removing capsid-damaged NoVs RNA, and photo-activatable dyes can enter non-infective capsids and bind to viral RNA under strong visible light conditions, thus preventing non-infective virus amplification [36]. Studies have shown that the PMA/RT-qPCR technique was able to remove approximately 46% of false positive results while being very effective in distinguishing the viability of NoVs in shellfish under high temperatures and long-duration conditions.

Microdrop droplet digital PCR (ddPCR) is a third-generation PCR technique that accomplishes detection by randomly distributing the reaction mixture of a sample into thousands of droplets and counting the number of negative and positive droplets with high sensitivity. Persson et al. used ddPCR and RT-qPCR to quantitatively detect NoVs in oysters and compared the results, which showed that ddPCR was more accurate than RT-qPCR in quantifying NoVs in oysters under reproducible conditions [74]. ddPCR is particularly suitable for risk assessment and outbreak analysis; however, the detection instruments are expensive, and the methods are time-consuming. Additionally, ddPCR involves many sample transfer steps, which increase the risk of sample contamination.

**Figure 2:**
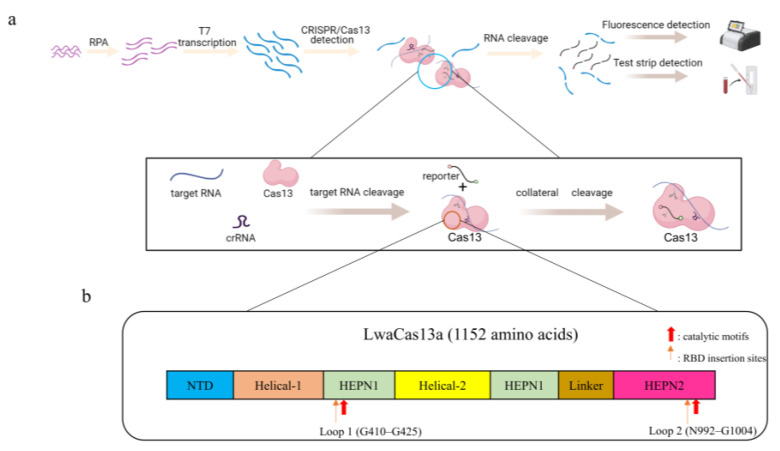
(**a**) Schematic diagram of the RPA-CRISPR/Cas13a assay principle. The samples are first amplified via RPA, and then a large amount of target RNA is generated by T7 transcriptase. The CRISPR/Cas13 system recognizes the target RNA by crRNA and then undergoes cis- and trans-cleavage to signal the RNA reporter. The detection results are visualized using fluorescence detection or test strip detection. (**b**) Domain organization of LwaCas13a. The catalytic motifs R474–H479 in HEPN1 and R1046–H1051 in HEPN2 are marked in red. The loop regions inserted by RBD G410–G425 (Loop 1) and N992–G1004 (Loop 2) are marked in orange.

Recently, molecular diagnostic technologies based on clustered regularly interspaced short palindromic repeat (CRISPR)-associated proteins (Cas) have shown great promise for various applications [75,76]. The CRISPR/Cas system is an acquired immune system in certain bacteria and archaea that recognizes specific targets via CRISPR RNA (crRNA) and induces the cleavage of Cas proteins after monitoring signals, such as fluorescence, indicating the detection of the target virus [76]. This technique provides a new direction for the development of molecular detection techniques for viruses. Duan et al. [77] combined CRISPR/Cas13a with recombinase polymerase amplification (RPA) and developed the RPA-CRISPR/Cas13a detection technique with a detection limit of 5 × 10^0^ copies/reaction, which achieved ultrasensitive and low-cost detection of NoV GII.4 (the assay principle and domain organization of LwaCas13a are shown in Figure 2). Therefore, CRISPR/Cas detection technology may become a powerful tool for detecting NoVs in oysters in the future.

## 6. Inactivation Methods

Using the strength of the binding ability of NoV-like particles (NoV VLPs) to porcine gastric mucin-coupled magnetic beads (PGM-MB) at different temperatures to study the thermal stability of NoVs, NoV VLPs were found to still bind to PGM-MB at 80 °C with heat treatment for 5 min; even when the temperature was increased to 100 °C, it took 5 s before NoV VLPs began to significantly lose the ability to bind with PGM-MB. To completely destroy NoVs, they need to be maintained at 100 °C for more than 15 min [78,79]. The nutritional value of the oyster itself can be severely damaged at such high temperatures for long periods; therefore, thermal processing (TP) may not be an ideal method to reduce the potential public health concerns associated with oysters. NTP can destroy pathogenic microorganisms to ensure food safety while retaining the flavor and nutritional value of the food itself and is widely used in fish processing [80]. NTP treatments for inactivating NoVs are shown in Table 2. NTP techniques include HHP treatment, irradiation and plasma treatment [81,82,83].

### 6.1. HHP

HHP is one of the most promising NTP methods for processing foods, in which virus inactivation is achieved by applying a high pressure of 100–600 Mpa [93]. In this process, pressurization occurs within the pressure chamber, with little risk of cross-contamination [94]. During the HHP treatment, the pressure is instantaneously and uniformly distributed throughout the oyster, ensuring that the viruses are treated uniformly. The adiabatic conditions of the process allow for only small temperature changes with increasing pressure (approximately 3 °C for every 100 MPa increase in pressure), thus maximizing the retention of the original flavor of oysters [95]. The inactivation effect of HHP on viruses depends on the substrate in which the virus is located. Typically, the treatment effect of HHP varies with different matrices; for example, among the three matrices of buffer, oyster homogenate and shucked oyster, the inactivation effect of HHP was greatest in the buffer, followed by oyster homogenates and shucked oysters [88]. A clinical trial on NoVs inactivation in oysters, in which oysters were treated for 5 min at 6 °C and 600 MPa, showed that none of the volunteers were infected with NoVs after consuming the oysters. These results indicated that HHP successfully inactivated NoVs in oysters under these conditions [96]. HHP inactivates the virus by denaturing the protein so that it cannot bind to the receptor on the surface of the host cell, thus reducing its infectivity [97]. NoVs are effectively inactivated by HHP; however, the pressure needs to be higher than 400 MPa to ensure that the concentration of NoVs is maintained at safe levels [90,98]. After HHP treatment, the shelf life of the food was prolonged, whilst the taste aspect was hardly affected and the change was much milder than that after conventional heat treatment [99]. After HHP treatment, the nutritional value and fresh taste of oysters are retained, and they are fuller and juicier in appearance than untreated oysters, which appeals to consumers.

#### Influencing Factors

The inactivation effect of HHP on pathogens is influenced by many factors, including processing parameters (pressure, temperature and time) and non-processing parameters (food matrix and pH) [100,101].

Usually, prolonging the pressurization time improves viral inactivation; however, increasing the pressure can result in a significant improvement in viral inactivation. As HuNoVs cannot be stably cultured in vitro at present, feline Culex virus (FCV) and MNV-1 are often used instead [102]. FCV showed a 2.8 log_10_ reduction in virus abundance after 20 min of treatment at 200 MPa and room temperature, and extending the time by 52 min resulted in only an additional 0.9 log_10_ reduction in the virus [103]. MNV-1 took 5 min to inactivate at 20 °C in the 350–450 MPa interval, and when the pressure reached 450 MPa, HHP treatment was able to inactivate 6.9 log_10_ of MNV-1 [7], demonstrating that increased pressure is the preferred method to improve the efficiency of HHP-based virus inactivation. Temperature also has a significant effect on the inactivation of NoVs by HHP. For MNV-1, a 350 MPa treatment at 30 °C for 5 min resulted in only a 1.2 log_10_ reduction in MNV-1, whereas lowering the temperature to 5 °C with the rest of the conditions unchanged achieved a 5.6 log_10_ reduction in the virus, improving the inactivation effect by nearly four-folds [104]. Sido et al. studied the effect of HHP on the inactivation of MNV-1 in shallots and showed that the inactivation of MNV-1 in shallots increased with the decrease in temperature [105]. Similar results were obtained by Li et al., who found that HuNoV GII.4 decreased by 2.9 log_10_ at 250 MPa and 1 °C for 2 min; and when the temperature increased to 21 °C, the inactivation decreased to 0.1 log_10_ [106]. This might be because low temperatures in HHP contribute to an increase in water density in the solvation cage, which causes severe shell protein deformation and enhances the inactivation effect [107,108].

pH is also one of the factors that affects the effectiveness of HHP in pathogen inactivation. FCV and MNV-1 are more easily inactivated by HHP in a neutral pH environment than at acidic pHs [109,110]. HHP treatment of MNV-1 for 2 min at 300 MPa, 4 °C, and pH 7.0 resulted in a reduction of 5.5 log_10_. At pH 4.0, with the remaining conditions unchanged, HHP only achieved a 2.5 log_10_ reduction in MNV-1 [100]. A study of FCV produced similar results, where HHP treatment of FCV for 1 min at 250 MPa, 20 °C, and pH 6.0 reduced FCV by 4.1 log_10_ but only by 0.5 log_10_ at pH 4.0 [109]. In addition, using receptor binding capacity as an indicator, it was found that the effect of pH on HuNoV GII.4 was similar to that on MNV-1, and both were difficult to inactivate in an acidic environment [89]. Considering that certain viruses are more resistant to an acidic environment, higher pressure needs to be applied to ensure the inactivation of such viruses when HHP treatment is performed on acidic food. The food matrix is another important factor affecting the inactivation effect of HHP. Carbohydrates, fats, proteins, ions, and other components of the food matrix can inhibit viral inactivation by HHP [111,112]. The inactivation effect of HHP on viruses can vary depending on the matrix [113]. For example, a food matrix treated with high-pressure HHP treatment may exhibit inferior viral inactivation compared to that of a food matrix treated with low-pressure HHP. For example, MNV in buffer and oysters were treated with HHP simultaneously, with a pressure of 200 MPa and 275 Mpa, respectively. The results showed a 2.7 log_10_ reduction in MNV in the buffer and only a 1.7 log_10_ reduction in oysters [88]. Therefore, before HHP treatment, parameters, such as pressure and time, must be adjusted according to the characteristics of the food matrix to achieve high-efficiency inactivation. It has also been reported that different strains have different susceptibilities to HHP treatment, even if they belong to the same genome. For example, the resistance of different strains of HuNoV GII to HHP are ranked as follows: GII.1 > GII.6 > GII.4. Many recent outbreaks caused by GII.1 and GII.6 may be related to their high resistance to the external environment [114].

### 6.2. Irradiation Processing

Compared to TP techniques, irradiation treatment has significant advantages in retaining the nutritional value of food products and has good removal rates for toxic and harmful substances [115]. In addition, irradiation treatment does not produce wastewater. It has no negative impact on the environment; therefore, it is an environmentally friendly technology [116]. Currently, several countries apply irradiation to various types of food treatment, including gamma irradiation, electron beam (e-beam) irradiation and X-ray irradiation.

Gamma rays are released during isotope-negative beta decay and can destroy pathogens by penetrating the interior of food products and inactivating them [117]. According to Park et al., interactions between gamma rays and nucleic acids include double (single) strand breaks, cross-link breaks and nucleotide degradation [118]. They used MNV-1 as a HuNoV surrogate and found that the expression of its major coat protein (VP1) gene decreased with increasing irradiation doses, whereas gamma rays were able to break covalent and non-covalent bonds, which play a critical role in protein structure. Similar results have been reported by Feng et al. [117]. They confirmed that the VP1 of MNV-1 is gradually degraded with increasing irradiation dose, and when the irradiation dose reached 22.4 kGy, the VP1 protein could not be detected. The number of intact virion structures exhibited the same phenomenon: the higher the irradiation doses, the lower the number, and they only existed as fragments at 22.4 kGy. Although gamma irradiation had a good inactivation effect on NoVs, irradiation doses >11.2 kGy were required to significantly inactivate the virus.

E-beam irradiation accelerates electron transfer using machines with high controllability and shorter processing times than gamma irradiation. E-beam irradiation often produces energy levels up to 10 MeV, which is approximately 10 times higher than that of gamma radiation. However, because of the limitation of the effective penetration depth, the removal effect of the e-beam irradiation is limited to the outer surface. DiCaprio et al. studied the effect of e-beam irradiation on the inactivation of HuNoV GII.4 and found that HuNoVs could not be detected by the PGM-MB binding assay when the irradiation dose reached 28.7 kGy [119]. Predmore et al. concluded that this was due to the electron beam degradation of viral proteins and RNA [120].

X-ray irradiation is another effective method for inactivating NoVs. Wu et al. showed that X-rays were effective in reducing MNV-1 in live oysters and found that even at the highest experimental dose of 5 kGy, X-ray irradiation could not kill live oysters and had no obvious effect on oyster viability, coloration, or other aspects [87].

### 6.3. Plasma Processing

Plasma is one of the states of matter and is defined as ionized gas [121]. Reactive oxygen species (ROS) and reactive nitrogen species (RNS) generated by gas ionization impede the entry of viruses into host cells and oxidize some amino acids in the VP1 structural domain (shell and protrusion structural domains), thereby destroying the viral coat protein, thus inactivating the virus in the matrix [122,123]. Cold atmospheric plasma (CAP) relies on various plasma sources, including jet, dielectric barrier discharge (DBD) plasma, and corona discharge plasmas [124]. Aboubakr et al. treated HuNoV GII.4 in lettuce leaves with DBD plasma-generated CAP for 5 min and observed a 2.6 log_10_ reduction in the virus [125]. Choi et al. further investigated the effect of this treatment method on HuNoV GII.4 in oysters using CAP generated by DBD plasma [126]. The results showed that HuNoVs were reduced by 1.05 log_10_ within 30 min. The oyster pH did not change significantly even after an experimental period of up to 1 h. Changes in pH are the simplest and most important indicator of changes in oyster quality [127]. Thus, this suggests that CAP reduces the infection level of HuNoVs without affecting the quality of the oyster and preserves the quality of the oyster to the maximum extent. There are large differences between the results of Aboubakr et al. and Choi et al. in that the same HuNoV GII.4 was treated with CAP, and the inactivation efficiency in the oyster substrate was smaller with longer treatment times, probably due to the presence of components in the oysters that have an inhibitory effect on the effect of CAP treatment.

## 7. Conclusions and Future Perspectives

Oysters are important marine food and major carriers of NoVs, and their safe consumption is critical. The filter-feeding nature of oysters allows NoVs present in the water column to enter and bioaccumulate in their digestive system. This bioaccumulation is a selective accumulation of NoV strains with multiple species of HBGA in the oyster body, with some initiation. Factors, such as temperature, precipitation, seasonal differences in ligand expression and water pollution, can cause differences in the bioaccumulation efficiency of NoVs in oysters; however, the primary influencing factors are unclear and require further research. The establishment of rapid, accurate and field-applicable NoVs detection methods will play an important role in the prevention of oyster-induced NoVs outbreaks. RT-qPCR detection technology is considered the gold standard for NoVs detection; however, it requires specialized detection instruments and professional personnel for operation and cannot determine whether NoVs are infectious. The new generation molecular detection technology CRISPR/Cas has great application prospects based on the specific recognition and cleavage of target sequences via CRISPR/Cas and the visualization of detection results with the help of fluorescent signal release. NoVs detection using CRISPR/Cas is particularly suitable for the on-site detection of NoVs in oysters because of its reliable results, simple operation and the lack of requirement for special instruments. The taste and freshness of oysters are greatly reduced by thorough heat treatment; therefore, NTP treatments need to be developed to inactivate NoVs present in oysters. NTP methods, such as HHP, irradiation treatment and plasma treatment, have good NoV removal effects, but they are limited by equipment and cost and have not been used commercially on a large scale. In addition to NTP methods to inactivate NoVs in oysters, avoiding NoVs contamination in oyster farming areas as much as possible by improving wastewater treatment processes should be considered.

Continued monitoring of molecular epidemiological data and continued research on the bioaccumulation mechanisms of different NoV strains in oysters will help in the development of specific drugs for the treatment of acute gastroenteritis caused by NoVs. At present, NTP treatments, such as HHP, can efficiently inactivate NoVs in oysters and have no negative impact on oyster appearance, taste, or nutritional value. Future studies should focus on improving these NTP treatment technologies to achieve large-scale applications and reduce the risk of NoVs spreading through oysters.

## Figures and Tables

**Table 1:** Detection rate of NoVs (GI and GII) in bivalve shellfish.

Area	Period	Species	GI (%)	GII (%)	References
Australia	2010–2011	Oysters	-	1.7	[27]
Italy	January 2013–July 2015	Mussels	3.0	26.0	[28]
		Oysters	-	1.0	
Thailand	August 2011–July 2012	Mussels	9.0	4.0	[29]
		Oysters	10.0	2.0	
		Clams	4.0	2.0	
UK	March 2015–March 2016	Oysters	15.7	14.0	[30]
China	March 2019	Oysters	6.6	21.2	[31]
Republic of Korea	Janurary 2011–September 2011	Oysters	7.8	15.7	[32]
Vietnam	October 2015–June 2016	Oysters	52.4	74.6	[33]
Moroccan Atlantic coast	November 2015–February 2017	Oysters	-	7.0	[13]
France	Janurary 2008–September 2008	Mussels	8.4	14.4	[34]
Singapore	2019–2020	Oysters	35.3	34.6	[35]

**Table 2:** Inactivation of NoVs via NTP technologies.

NTP Technologies	NoV Types	Processing Conditions	Initial Concentration	Reduction (%)	References
e-beam irradiation	MNV-1	5 kGy	4.9 log PFU/mL	12	[84]
e-beam irradiation	MNV-1	7 kGy	6.06 log_10_ PFU/mL	30	[85]
X-Ray irradiation	MNV-1	4 kGy	6.3 log PFU/mL	59	[86]
X-Ray irradiation	MNV-1	1 kGy	4.3 log PFU/g	16	[87]
		2 kGy		25	
		3 kGy		35	
		4 kGy		42	
HHP	MNV-1	275 MPa, 2 min, 0 °C	4.8 log PFU/oyster	2	[88]
		275 MPa, 5 min, 0 °C		42	
HHP	MNV-1	350 MPa, 2 min, 4 °C	5.5 log_10_ PFU/g	36	[89]
HHP	HuNoV GII.4	300 MPa, 5 min, 6 °C	4 log_10_ units	72	[90]
		400 MPa, 5 min, 6 °C		90	
	HuNoV GI.1	300 MPa, 5 min, 6 °C		17	
		400 MPa, 5 min, 6 °C		32	
HHP	HuNoV GII.4	350 MPa, 2 min, 0 °C	4–5 log_10_ units	>84	[91]
		350 MPa, 2 min, 25 °C		72–90	
	HuNoV GI.1	500 MPa, 2 min, 0 °C		>86	
		500 MPa, 2 min, 25 °C		16–20	
HHP	HuNoV GII.4	350 MPa, 2 min, 21 °C, pH 4	6 log_10_ units	30	[92]
	HuNoV GI.1	550 MPa, 2 min, 21 °C, pH 4		18	
HHP	HuNoV GII.4	200 MPa, 5 min, 5 °C	8.95 × 10^5^ log_10_ genome copies	89	[80]
	HuNoV GI.5		4.39 × 10^5^ log_10_ genome copies	85	

## Data Availability

The data presented in this study are available on request from the corresponding author.
